# Analysis of Socio-Demographic Profiles of Suicidal Hanging Cases to Formulate a Preventive Strategy: An Autopsy-Based Study Conducted at a Tertiary Care Hospital in the North-East Region of India

**DOI:** 10.7759/cureus.42483

**Published:** 2023-07-26

**Authors:** Samarendra Barman, Kaustav Kumar Bairagi

**Affiliations:** 1 Department of Forensic Medicine and Toxicology, All India Institute of Medical Sciences, Guwahati, IND

**Keywords:** north east region of india, preventive strategy, socio-demographic characteristics, autopsy, hanging, suicide

## Abstract

Introduction

Hanging is a common method of suicide in India and worldwide. Analyzing the socio-demographic characteristics to formulate a preventive strategy to reduce the incidence of suicide by hanging is often considered a low-cost but effective intervention for developing countries like India. The present study reports on preventive measures based on socio-demographic data of suicidal hanging cases brought for autopsy in a city in Northeast India. There is no scientific literature originating from the Northeast region of India that stresses on the preventive aspect of suicidal hanging cases to date.

Methods

This is an observational study based on retrospective data. Data related to socio-demographic characteristics were collected from all the suicidal hanging cases reported for medicolegal autopsy at a tertiary care hospital in Dibrugarh, a city in Northeast India, from June 2012 to June 2013.

Results

A total of 1241 cases were brought for autopsy, out of which 70 (5.64%) cases were determined to be death due to suicidal hanging. Male victims accounted for the majority of the cases, and most victims were in their third decade of life. Additionally, various other factors, such as marital status, occupational status, place of occurrence, psychiatric history, pregnancy status, and selection of suspension point, were studied.

Conclusion

Socio-demographic data generated from various research studies can play a crucial role in the early identification of vulnerable individuals and enable the prompt delivery of mental health services and other measures of interventions. Implementing policy-based strategies, such as restricting access to means and materials used in hanging, can have a positive impact on overall outcomes. Additionally, involving electronic or print media to de-popularize hanging as a clean and painless method proves to be another effective intervention.

## Introduction

Hanging is a frequent method of committing suicide in India and worldwide. There is a rising incidence of hanging cases in different parts of the world as many believe this is a simple, certain, clean, and painless method of self-destruction [1]. According to the National Crime Records Bureau (NCRB) data, in 2021, 57% of the total suicides in India occurred by hanging, while 25.1% happened by poison consumption [2]. This rising trend can be seen in many developed countries too. However, there are marked differences in suicidal behaviour between developed and developing countries. A 2019 World Health Organization (WHO) report stated that mental disorders, particularly depression, and alcohol abuse are the leading causes of suicide in high-income countries [3]. In the UK, as per the NCRB data, family problems, unemployment, and poverty are the most common reasons for committing suicide [2].

With the rising incidence, suicidal hanging has become a significant socio-economic and public health burden in India. The government is urging research planners to take urgent preventive measures to curb this menace. One cost-effective approach to address this situation in a particular region is to gather various socio-demographic data associated with suicidal hanging cases originating from that specific geographical area. This data can be used to formulate a preventive strategy.

In line with this objective, the present study is conducted in a tertiary care hospital in Dibrugarh, a city in the North-East region of India. The aim is to identify vulnerable members of the target population and determine areas for intervention. Notably, no records of such a study have been found from the North-East region of India until now.

## Materials and methods

Objectives 

The aim of the study was to analyze the socio-demographic characteristics of hanging cases autopsied during the study period and to formulate preventive strategies based on the socio-demographic characteristics. 

Study design

This is an observational study based on the retrospective data generated from the autopsied cases. 

Study samples and site

The study sample includes all the cases of suicidal hanging autopsied in a tertiary care hospital in Dibrugarh during the period from June 2012 to June 2013. The study population was natives of urban and rural areas of Dibrugarh, a region in North-East India.

Data collection and ethical permission

Data on socio-demographic characteristics are collected on the basis of police inquest reports, magistrate inquest reports, statements of the investigating officers who investigated the scene of death, accounts from close relatives, and eyewitness accounts. The data were collected after approval by the Institute Ethics Committee (Ref No: AMC/IEC/PG/40/2012), and the Research Cell of the current institute has been informed through letter no AIIMS/Guwahati/FMT/55/23 about the intention to publish.

Data analysis

All data were manually entered into a Microsoft Excel sheet (Microsoft Corporation, Redmond, Washington, United States) and were statistically analyzed by presenting the data in the form of appropriate tables and computing the frequency and percentages. Due to the nature of the assessment, no averages or correlations were calculated, and thus, p-values are not relevant to the study parameters.

## Results

During the study period, a total of 1241 cases had been brought for medico-legal autopsy, and among them, 70 cases (N) were due to suicidal hanging. Male victims accounted for 71.43% of the cases, with a male-female ratio of 2.5:1, as shown in Table 1.

**Table 1:** Sex-wise distribution of hanging cases (N = 70)

Sex	Frequency (n) and Percentage (%)
Male	50 (71.43)
Female	20 (28.57)

The majority of hanging cases belonged to the age group of 21-30 years, accounting for 32.86% of the cases, followed by the 11-20 years age group with 21.43% of the cases. The least number of cases were observed in the age groups of below 10 years, 61-70 years, and 81-90 years, each accounting for 1.43% of the total cases. The results are presented in Table 2.

**Table 2:** Age-wise distribution of hanging cases (N = 70)

Age In years	Male	Female	Frequency (n) and Percentage (%)
Upto 10	00	01	01 (1.43)
11-20	05	10	15 (21.43)
21-30	17	06	23 (32.86)
31-40	10	02	12 (17.14)
41-50	09	00	09 (12.86)
51-60	03	00	03 (4.29)
61-70	01	00	01 (1.43)
71-80	04	01	05 (7.14)
81-90	01	00	01 (1.43)

Table 3 shows that the incidence of hanging was higher among married individuals, accounting for 64.28% of the cases, as compared to unmarried individuals, which had 32.86% of the cases.

**Table 3:** Marital status-wise distribution of cases (N= 70)

Marital Status	Male	Female	Frequency (n) and Percentage (%)
Married	37	08	45 (64.28)
Unmarried	13	10	23 (32.86)
Widow/er	00	02	02 (02.86)
Divorcee	00	00	00 (00)

Table 4 shows the occupation-wise distribution of cases, revealing that the majority belonged to daily wage earners with 28.57% of the cases, followed by the unemployed group with 18.57% of the cases. Students accounted for 17.14% of the cases, while service holders and the retired group accounted for 5.71% of the cases.

**Table 4:** Occupation-wise distribution of cases (N = 70)

Traits	Frequency (n) and Percentage (%)
Daily wage earner	20 (28.57)
Unemployed	13 (18.57)
Student	12 (17.14)
Self-employed	08 (11.43)
Homemaker	07 (10.00)
Service holder	04 (5.71)
Retired	04 (5.71)
Cultivator	02 (2.87)

Complete hanging accounted for 71.43% of the cases, compared to 28.57% of cases for partial hanging. The incident occurred inside the victim's own house in 68.6% of cases, while in 7.14% of cases, the victim chose a relative's house to commit suicide. The results are shown in Table 5.

**Table 5:** Place of occurrence-wise distribution of cases (N = 70)

Places	Frequency (n) and Percentage (%)
Own house	48 (68.6)
Outside	11 (15.7)
Relative’s house	5 (7.14)
Hotel room	3 (4.28)
School	3 (4.28)

Table 6 displays the distribution of cases according to the selection of the suspension point. In all household cases, the suspension point was located either on the ceiling fan, ventilator grill, or roof beam. Among these cases, 30% of victims used the ceiling fan as a suspension point, followed by 23% of cases using the ventilator grill.

**Table 6:** Selection of suspension point (N = 70)

Traits	Frequency (n) and Percentage (%)
Ceiling fan	21 (30)
Ventilator grill	16 (23)
Roof beam	12 (17)
Tree branch	8 (12)
Window grill	7 (10)
Telephone pole	3 (4)
Furniture handle	3 (4)

In 11.4% of cases, there was a documented history of prior suicidal attempts. In 8.6% of cases, the victims had a documented history of major depression. Among female victims, 20% were pregnant at the time of the act.

## Discussion

Observation

In the present study, hanging accounted for 5.64% of total cases and was found to be one of the most common methods of committing suicide, consistent with Indian as well as global data [4,5]. Male victims outnumbered female victims, with a male-female ratio of 2.5:1. The predominance of male cases in suicidal hanging is a recurring finding observed in various other studies [6-8]. While there is no definitive explanation for why the incidence of hanging is higher in males than females, cultural and societal factors may contribute to this gender difference. For instance, traditional masculine ideals that emphasize self-reliance, stoicism, and strength may make it challenging for men to seek help or express their emotions. Consequently, some men may internalize their feelings of distress and hopelessness, potentially increasing their risk of suicide.

Most of the victims in the study belong to the age group of 11-40 years, which is consistent with observations from various other studies [6,9,10]. This trend can mainly be attributed to the fact that the age group 11-40 years is the most active and crucial period of life, and there are significant stress and strain associated with this stage. The zeal and urge to excel in this highly competitive world, financial instability, job insecurity, disillusionment, and frustration may lead to depression during this phase of life. Additionally, conflicts related to marriage, such as dowry harassment and marital disputes, are some of the major social issues that play a crucial role in the development of depression and suicidal thoughts among individuals in this productive age group.

The present study observes a higher number of suicidal hanging cases among married individuals compared to unmarried individuals. This finding aligns with the NCRB report of 2014, which showed that 66% of all suicides were committed by married people [11], while only 21% were unmarried. Similar observations have been noted in a few other studies as well [12]. Although no specific reason can be definitively attributed to this finding, possible explanations may include family issues, broken marriages, financial problems, and psycho-somatic illnesses, among others.

In the present study, 20% of the female victims were found to be pregnant at the time of committing the act. This observation aligns with one study that reported hanging as the most common method of committing suicide among pregnant women [13]. Numerous researchers have acknowledged that suicidal ideation is prevalent among pregnant women. Although the exact reason is still unknown, various plausible hypotheses have been investigated. Pregnancy can be challenging and stressful for many women, especially if associated with risk factors such as unplanned pregnancy, a history of childhood abuse, intimate partner violence, pre-existing vulnerability (e.g. family history of suicide), financial difficulties, or other sources of stress [14]. These factors can increase the likelihood of depression and other mental health issues, subsequently increasing the risk of suicidal thoughts and behaviours.

Regarding socio-economic status, the present study revealed that daily wage earners accounted for 28.57% of cases, while the unemployed group comprised 18.57% of the victims, making up the majority. Several other studies have observed similar findings, where the majority of victims belong to the low socioeconomic group [15]. In one study, it has been stated that suicide in India is more closely associated with poverty than mental illness [16]. This can be attributed to a positive correlation between low socioeconomic status and depression and suicidal thoughts.

It has been found that the majority of the victims in the present study hanged themselves in their own homes, accounting for 68.6% of cases. In 30% of cases, the victims preferred a ceiling fan as a point of suspension, followed by 17% of cases where a high-lying roof beam was chosen. A secluded environment with suitable aids provides sufficient preparation for suicide by hanging, allowing the victim to act relatively quickly, impulsively, and without being noticed by others.

Ceiling fans are a common suspension point for hanging because they are typically securely installed into the ceiling structure and can support a significant amount of weight [17,18]. Their sturdy construction and central location make them a practical choice for hanging. Another reason for choosing a high suspension point, such as a ceiling fan, for hanging is to achieve complete suspension.

Complete hanging accounted for 71.43% of cases in the present study. This finding is consistent with various other studies [19,4]. The increased number of complete hanging cases may be due to the common belief among those who attempt suicide that death is more rapid in complete hanging, as there is a more constricting force with full suspension of the body. If a person is not fully suspended, the weight of their body may not be sufficient to cause the neck to break, or the noose may not tighten enough to cut off the blood supply to the brain or the air supply to the lungs. This can result in a prolonged and painful death, which is not the desired outcome in cases of hanging.

 Preventive strategy

Seeking effective intervention for suicide prevention represents an important public health task. Restricting access to means of suicide is a major preventive measure together with strategies aimed to identify and prevent suicidal acts in individuals at risk [20]. Hanging is a very simple but effective method of self-destruction, and materials required for its execution can be readily obtained from households without notice. For a majority of victims, the idea to commit suicide comes up suddenly, and taking away the easily accessible means may thwart the impulsive action and allow the suicidal individual to rethink their decision. 

Ideally, restriction of access to means is most effective in controlled environments such as prison cells, hospitals, police custody, etc., where the authorities have control over the rooms and can modify potentially lethal variables [21]. Materials used for hanging, such as ropes, belts, or any ligature materials, can be removed from the rooms of high-risk individuals. Structures must be constructed, and furniture must be placed in a way that makes the process of committing and accomplishing hanging difficult for potential victims.

A ceiling fan, which is a very common suspension point for many potential victims, can be equipped with an anti-suicide fan rod [22]. When someone tries to hang with an anti-suicide fan rod, both the fan and the victim fall on the ground safely due to the spring action of the fan rod. This type of ceiling fan is designed to fall when the loading weight exceeds a certain threshold weight. Such equipment is widely marketed after being tested in the Government of India’s MSME (Ministry of Micro, Small, and Medium Enterprise) lab. Another effective measure concerning ceiling fans is to replace them with wall-mounted fans or table fans. In many places in India, hostel authorities have designed iron grills to cover ceiling fans as a hanging preventive measure.

Another approach is to make the house walls free of any outward projections and remove any potential points of suspension. Preferred points of suspension must be identified and either removed or properly secured. Household ventilators and windows can be designed without grills.

While implementing these measures, it is essential to understand that complete suspension is not an absolute requirement for successful hanging. Help from an external expert can be sought to design in-house suicide prevention structures. In a controlled environment, in-house working staff should be adequately trained to identify high-risk individuals, and an effective response mechanism should be in place for those high-risk areas [23].

Mental health support for high-risk individuals is another effective preventive strategy against suicidal hanging [23]. Mental health support services and resources can help individuals who may be experiencing suicidal thoughts or feelings. Vulnerable individuals should not be allowed to stay alone. In hostels, students should not be given single rooms; instead, the hostel authority must encourage all boarders to share rooms. Individuals from the high-risk category must be identified based on medical records, prior history, and other socio-demographic characteristics, and they should be encouraged to seek psychological counselling.

Psycho-social correlates should be addressed separately for males and females to identify the population at risk. Improved pre-hospital and acute medical management of victims of near hanging is another key area that can have an impact on the current death toll [23]. Consensus guidelines on the management of near-hanging cases should be formulated and made available to ambulance and emergency department staff.

Another effective method to control this global menace would be through population-based initiatives to reduce the popularity of this method. This can be achieved by working with the media to reduce the fictional portrayal of suicidal hanging in movies or theatres to de-popularize the method. General suicide prevention measures for example regular suicidal risk assessment of the target group in ambulatory therapy, staff training program to identify high-risk people, setting up of suicide prevention centres, raising awareness among the public, etc. must be promoted and expanded to reduce the incidence. 

Limitations

The study sample is limited to cases received by the Forensic Medicine Department of the tertiary care hospital in Dibrugarh, based on the inclusion criteria. This limitation may result in the sample not being fully representative of all the suicidal hanging cases in the northeast region of India. Additionally, information related to the socio-demographic profiles of the victims was obtained from their legal guardians, next of kin, police reports, etc., which may be subjected to inconsistencies and biases. It is essential to consider these limitations while interpreting the results and formulating preventive strategies. The authors believe that incorporating larger study samples from multiple centres in the northeast region of India and collecting data on the victims through scene examinations and standardized questionnaires and interviews can provide a better insight into the problem and lead to more effective preventive strategies.

## Conclusions

The study revealed that preventive measures should be comprehensive and multi-disciplinary for a meaningful impact on society. A policy-based universal strategy should be adopted and directed towards the most vulnerable populations. Adolescent and young adult individuals should receive the maximum focus of intervention as they are the most vulnerable due to their tender age, heightened expectations, and societal demands. In a developing country like India, where socioeconomic factors play an important role in the overall well-being of the people, the government should come up with an effective strategy to mitigate issues related to unemployment, poverty, inequalities, etc., particularly among vulnerable individuals. Other high-risk groups, such as pregnant women, people with documented mental illnesses like depression, and individuals with a history of prior suicide attempts, need to be promptly identified and given timely access to mental health support services.

Society should be sensitised through electronic or print media to openly discuss these issues among all members and public health groups. Families of vulnerable individuals should be motivated to communicate and spend more time with those at risk. Another effective measure is restricting access to means and materials for hanging and implementing structural modifications in the households of vulnerable individuals. This is particularly effective in various controlled environments such as hospitals, prison cells, hostels, etc.
